# Colonic phosphocholine is correlated with *Candida tropicalis* and promotes diarrhea and pathogen clearance

**DOI:** 10.1038/s41522-023-00433-0

**Published:** 2023-09-04

**Authors:** Xihong Zhou, Yiwen He, Jingqing Chen, Xia Xiong, Jie Yin, Jing Liang, Can Peng, Chunxia Huang, Guiping Guan, Yulong Yin

**Affiliations:** 1 Key Laboratory of Agro-ecological Processes in Subtropical Region, Institute of Subtropical Agriculture, the Chinese Academy of Sciences, Changsha, China; 2 College of Advanced Agricultural Sciences, University of Chinese Academy of Sciences, Beijing, China; 3 School of Stomatology, Changsha Medical University, Changsha, China; 4 Hunan Provincial Key Laboratory of Animal Intestinal Function and Regulation, College of Life Sciences, Hunan Normal University, Changsha, China; 5 Laboratory Animal Center of the Academy of Military Medical Sciences, Beijing, China; 6 College of Animal Science and Technology, Hunan Agricultural University, Changsha, China; 7 College of Bioscience & Biotechnology, Hunan Agricultural University, Changsha, China

**Keywords:** Microbiome, Cellular microbiology

## Abstract

Diarrhea is characterized by alterations in the gut microbiota, metabolites, and host response to these changes. Studies have focused on the role of commensal bacteria in diarrhea; however, the effect of fungi on its pathogenesis remains unexplored. Here, using post-weaned piglets with or without diarrhea, we found an unexpected decrease in the abundance of *Candida tropicalis* in diarrheal piglets. We also observed increased accumulation of reactive oxygen species (ROS) and the formation of neutrophil extracellular traps (NETs) in the colonic tissues of diarrheal piglets. Using dectin-1-knockout mice, we found that the over-accumulation of ROS killed *C. tropicalis* by promoting NET formation, which was dependent on dectin-1. The decreased abundance of *C. tropicalis* resulted in reduced phosphocholine consumption. Then, colonic phosphocholine accumulation drives water efflux by increasing cAMP levels by activating adenylyl cyclase, which promotes the clearance of pathogenic bacteria. Collectively, we demonstrated that phosphocholine is correlated with colonic *C. tropicalis* and promotes diarrhea and pathogen clearance. Our results suggest that mycobiota colonizing the colon might be involved in maintaining intestinal metabolic homeostasis through the consumption of certain metabolites.

## Introduction

The gastrointestinal tract is one of the most complex systems in the body and plays a fundamental and central role in the absorption and excretion of fluids and nutrients. Diarrhea can occur due to dysfunctions in these abilities and accounts for nearly 4% of all human deaths every year, mostly in children, according to the World Health Organization^1^. Accordingly, diarrhea remains one of the major causes of childhood mortality globally^2^, since children have an immature digestion and absorption system and a vulnerable immune system. However, studies on infant and child diarrhea are subject to restrictions including those related to ethical issues and sample collection. Despite decades of research on the induction and development of diarrhea, the underlying molecular mechanisms remain largely unknown. Pigs have been used as models for studying human diseases because they are metabolically and genetically similar to humans^3,4^. Importantly, post-weaned piglets are anatomically and physiologically similar to children^5^. After weaning, piglets are exposed to a variety of stresses, including alterations in the environment and diet, and attacks from exogenous pathogenic bacteria^6^. Piglets exposed to these conditions can easily develop diarrhea, making them a perfect model for studying this condition.

Gut microbiota is closely involved in the occurrence and development of diarrhea. Patients with diarrhea-predominant irritable bowel syndrome show increased levels of Enterobacteriaceae^7^, and post-weaned piglets with diarrhea are characterized by a lower abundance of *Bacteroides*^8^. Additionally, the miRNA–bacteria and mucin O-glycan–bacteria axes have been proven to be related to diarrheal pathogenesis^6,9^. However, little is known about whether the mycobiota, as part of the intestinal microbiota, is involved in the regulation of diarrheal diseases. Recently, patients with inflammatory bowel diseases were found to exhibit alterations in their mycobiota^10–12^, especially with a high abundance of *Malassezia restricta*, a common skin-resident fungus that was found to exacerbate colitis by promoting inflammatory cytokine production in mice^13^. Taken together, these data suggest that intestinal fungi may play an important role in the maintenance of intestinal functions and disease pathogenesis. However, the role of these changes in these diseases remains unclear.

To determine whether and how intestinal fungi might be involved in the occurrence of diarrhea in post-weaned piglets, we examined the mycobiota composition in the feces of piglets with diarrhea immediately after weaning and healthy control piglets using a fungal ITS sequencing approach. Unexpectedly, we observed a decrease in the abundance of *Candida tropicalis* in diarrheal piglets, which was negatively correlated with increased phosphocholine levels. We found that the accumulation of reactive oxygen species (ROS) in diarrheal piglets due to post-weaning stress killed *C. tropicalis* by promoting the formation of neutrophil extracellular traps (NETs), which was dependent on *dectin-1* gene expression. Furthermore, decreased abundance of *C. tropicalis* resulted in reduced phosphocholine consumption. Consequent phosphocholine accumulation in the colon was found to drive water efflux by increasing cAMP levels via activating adenylyl cyclase, which promoted the clearance of pathogenic bacteria. Collectively, we suggest that the mycobiota colonizing the colon are involved in maintaining intestinal metabolic homeostasis via the consumption of certain metabolites.

## Results

### Mycobiota composition is altered in the feces of piglets with diarrhea

**Fig. 1:**
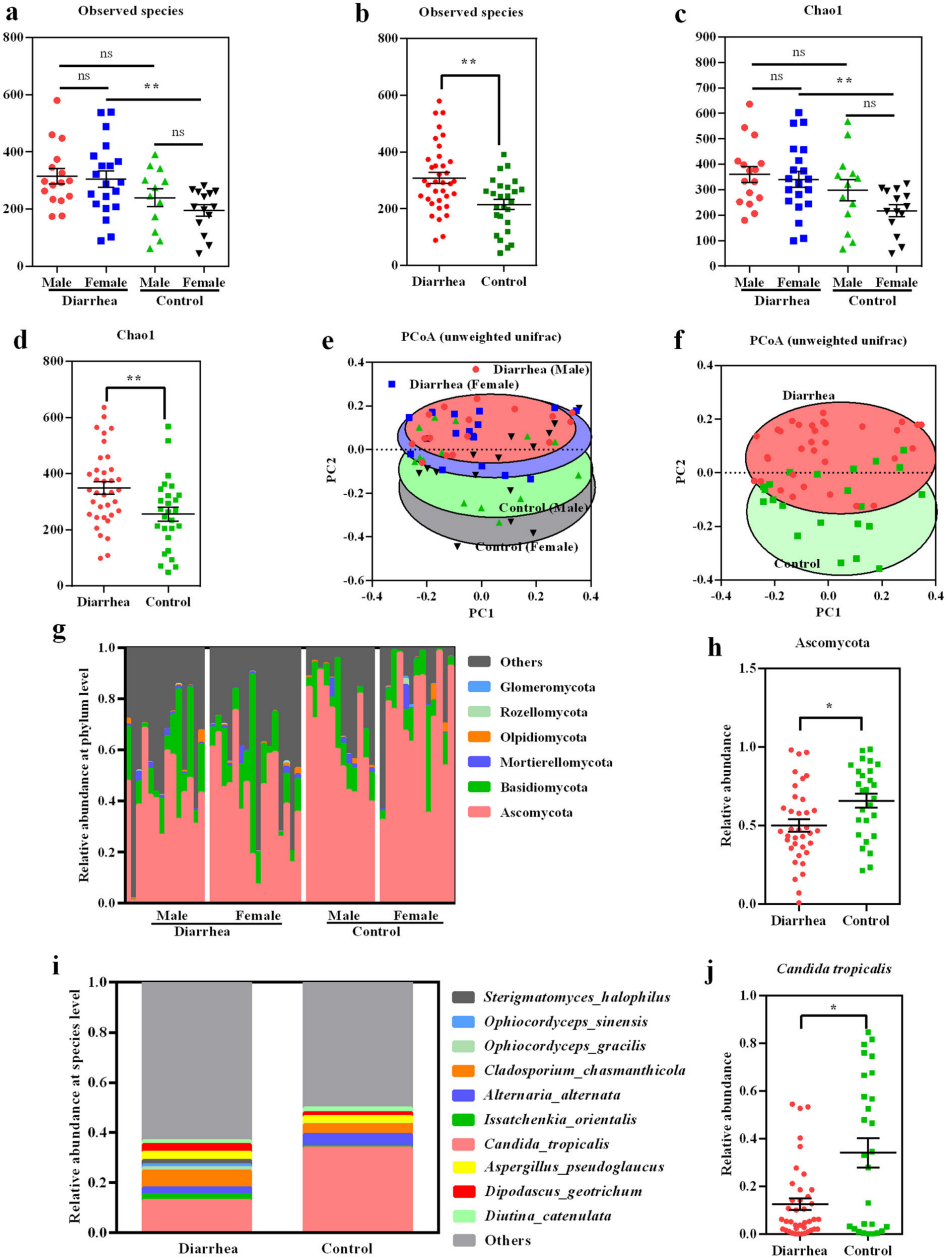
Mycobiota composition is altered in the feces of piglets with diarrhea. **a**, **b** Observed species; **c**, **d** Chao1 index; **e**, **f** PCoA based on unweighted UniFrac distance; **g** Relative abundance of fungi at the phylum level; **h** Relative abundance of Ascomycota; **i** Relative abundance of fungi at the species level; **j** Relative abundance of *C. tropicalis*. Data were either analyzed by two-tailed Student’s t-test or by factorial ANOVA. Data were presented as mean ± SEM. *P < 0.05, **P < 0.01. ns, not significant, *n* = 38 for diarrheal piglets (*n* = 18 for diarrheal male, *n* = 20 for diarrheal female); *n* = 27 for control piglets (*n* = 13 for control male and *n* = 14 for control female).

We collected fecal samples from thirty-eight diarrheal piglets and twenty-seven healthy control piglets to explore their differences in mycobiota by sequencing the fungal ITS1/2 regions. The results showed that the observed species and Chao1 indices were significantly higher in diarrheal piglets than in healthy control piglets, but no difference was observed between the sexes (Fig. 1a–d). Principal coordinate analysis (PCoA) based on the unweighted UniFrac distance showed that the fungal structure in diarrheal piglets was different from that in control piglets (Fig. 1e, f). To identify specific alterations in fungi, we analyzed changes in the composition of fecal fungal populations. Overall, Ascomycota and Basidiomycota were the two major fungi in the piglets at the phylum level (Fig. 1g). The results showed that the relative abundance of Ascomycota was significantly lower in diarrheal piglets than in control piglets (Fig. 1h). Furthermore, *Candida tropicalis* was the most abundant species in the feces of piglets, and its abundance was significantly lower in diarrheal piglets than that in control piglets (Fig. 1i, j).

### Metabolite composition is altered in the feces of piglets with diarrhea

**Fig. 2:**
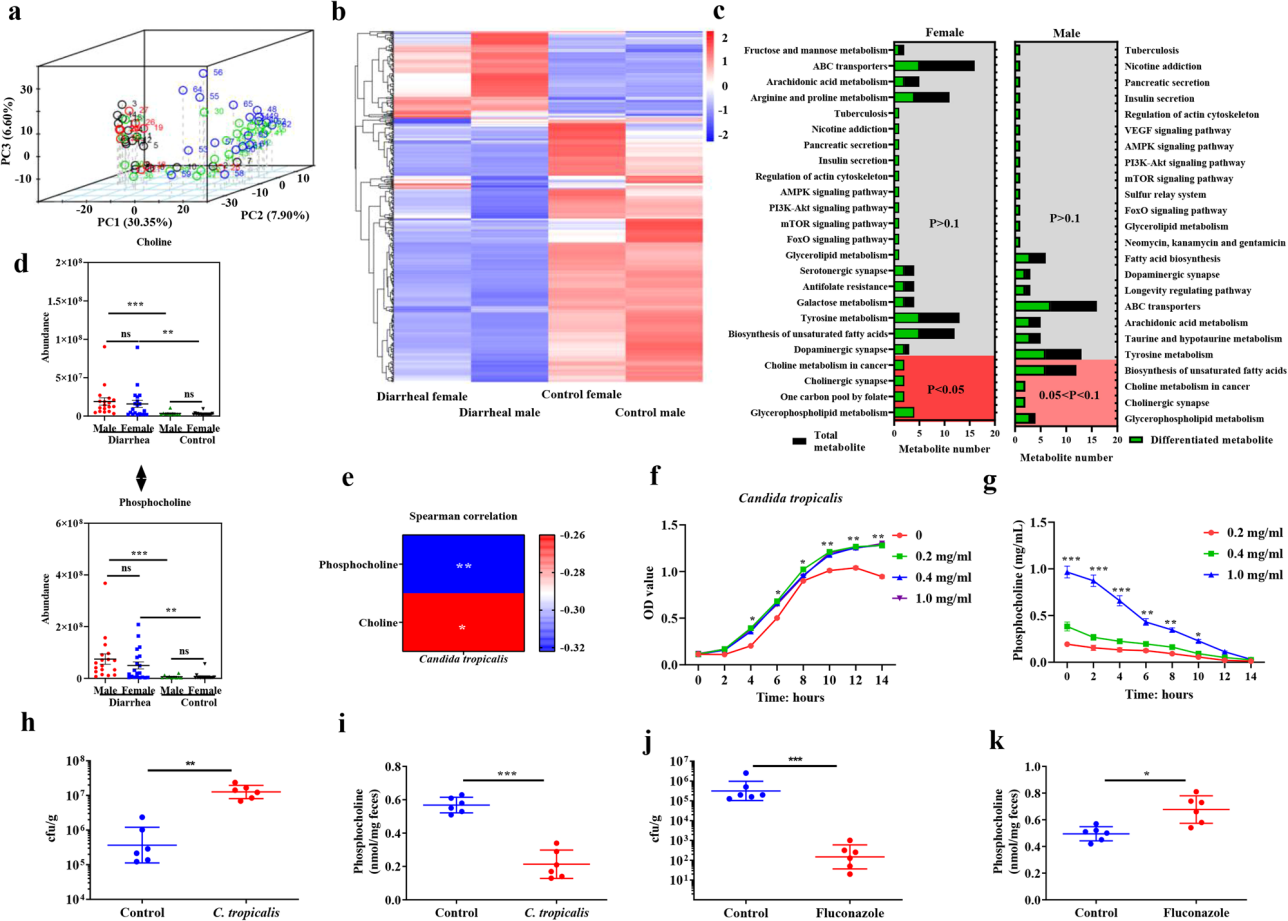
Decreased consumption by *C. tropicalis* is responsible for phosphocholine accumulation in diarrheal piglets. **a** PCA plot (dark, healthy female; red, healthy male; green, diarrheal female; blue, diarrheal male); **b** Heatmap showing the changes of metabolites; **c** KEGG analysis revealing the affected pathways; **d** Relative abundance of choline and phosphocholine (The data were analyzed by factorial ANOVA; *n* = 18 for diarrheal male, *n* = 20 for diarrheal female, *n* = 13 for control male and *n* = 14 for control female.); **e** Correlation of *C. tropocalis* level with the abundance of choline and phosphocholine, as determined by Spearman correlation analyses. **f** Addition of different contents of phosphocholine on *C. tropocalis* growth. **g** The alteration of phosphocholine content in the culture medium with time (The data were analyzed with one-way ANOVA, *n* = 3). Weaned piglets were orally gavaged with PBS or with 1 × 10^8 ^*C. tropicalis*, and then *C. tropicalis* **h** and phosphocholine content **i** in the feces were determined at day 7; Weaned piglets were either fed a basal diet or a basal diet supplemented with 0.1% fluconazole, and then *C. tropicalis* **j** and phosphocholine content **k** in the feces were determined at day 7 (Data were analyzed by two-tailed Student’s t-test, *n* = 6). Data were presented as mean ± SEM. *P < 0.05, **P < 0.01, ***P < 0.001. ns not significant.

To assess the metabolic alterations between healthy control and diarrheal piglets, an untargeted metabolomics approach was used on fecal samples. The principal component analysis (PCA) plot showed that metabolite profiles were distinctly different between diarrheal and healthy control piglets, whereas sex had no effect on the metabolite profiles (Fig. 2a). Further analysis indicated dramatic changes in the metabolites between diarrheal and healthy control piglets, as shown in the heatmap (Fig. 2b). KEGG analysis revealed that the significantly affected pathways were choline metabolism and glycerophospholipid metabolism (Fig. 2c). Among the metabolites enriched in these two pathways, choline and phosphocholine levels were significantly higher in diarrheal piglets than in the control piglets (Fig. 2d).

### Decreased consumption by *C. tropicalis* is responsible for phosphocholine accumulation

Correlation analyses of *C. tropicalis* with choline and phosphocholine levels showed that this species was negatively correlated with both metabolites (Fig. 2e). To further explore whether *C. tropicalis* was affected by these two substances, we cultured *C. tropicalis* with either choline or phosphocholine. The results showed that doubling times of *C. tropicalis* in media with different phosphocholine levels were similar, but differences were observed in cell density during the stationary phase (Fig. 2f). However, choline did not affect *C. tropicalis* growth (Supplementary Fig. 1). We further found that phosphocholine levels in the culture medium decreased over time (Fig. 2g), suggesting that *C. tropicalis* consumed phosphocholine for growth.

To explore whether host metabolism is also responsible for the accumulation of phosphocholine, the activity of choline kinase, which catalyzes the production of phosphocholine, was measured. However, the choline kinase activity did not change in the colon (Supplementary Fig. 2a). In addition, phosphocholine content in the colon (Supplementary Fig. 2b) was not altered. These data indicate that the accumulation of phosphocholine in the feces of diarrheal piglets is not caused by host choline metabolism. To further explore whether *C. tropicalis* abundance affects the intestinal accumulation of phosphocholine, weaned piglets were orally gavaged with PBS or *C. tropicalis*. We found that *C. tropicalis* abundance increased (Fig. 2h), whereas phosphocholine content decreased (Fig. 2i) in the feces of piglets administered *C. tropicalis*. The piglet diet was then supplemented with fluconazole, which targets *Candida* species. We found that *C. tropicalis* abundance was significantly decreased (Fig. 2j), whereas phosphocholine content was significantly increased (Fig. 2k) in the piglet feces. These results indicate that intestinal *C. tropicalis* abundance negatively affects phosphocholine content in weaned piglets.

### Accumulated ROS kill *C. tropicalis* via promoting the formation of NETs

**Fig. 3:**
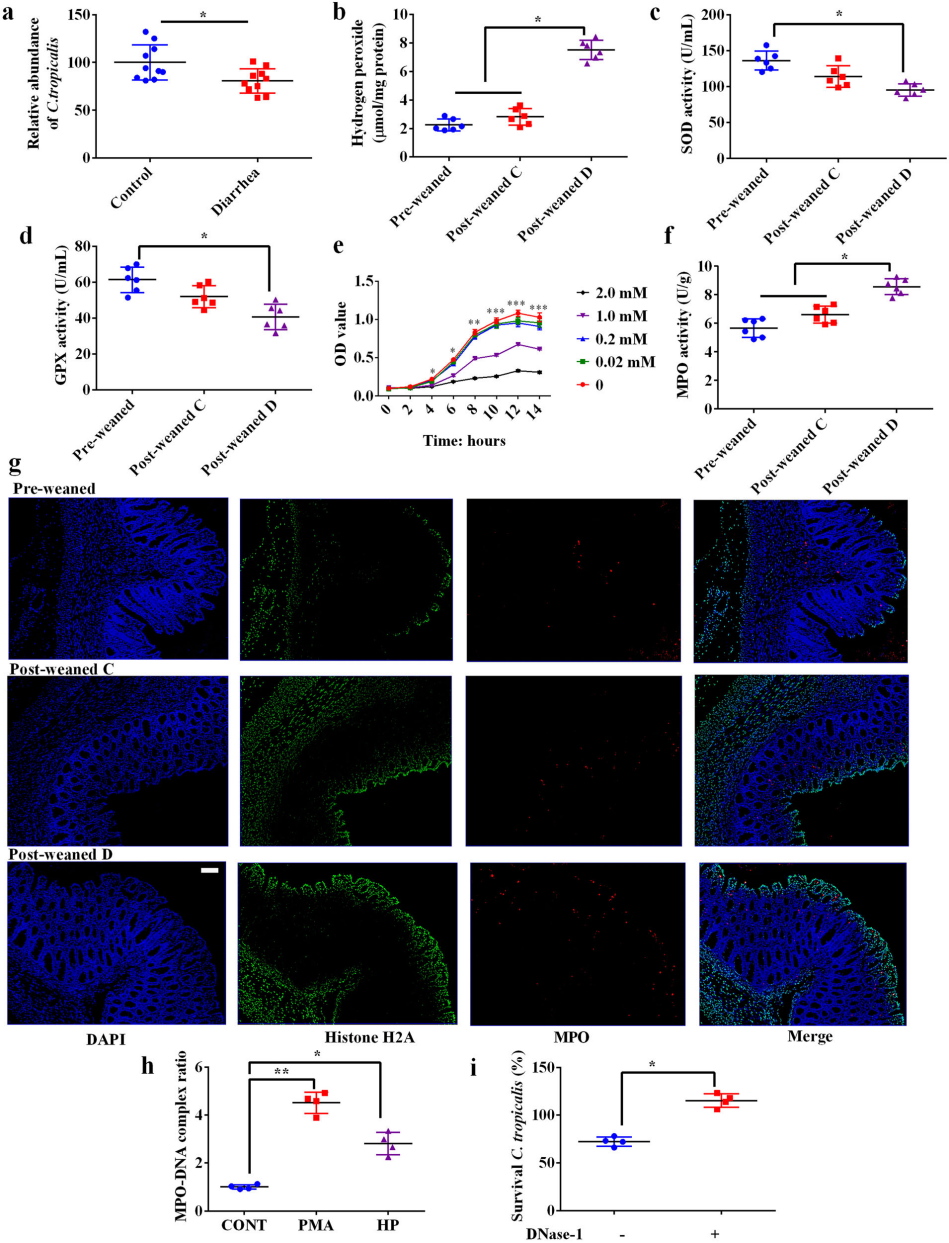
Accumulated ROS kill *C. tropicalis* via promoting the formation of NETs. **a** Relative abundance of *C. tropocalis* in the colonic mucosa of healthy control piglets relative to those of diarrheal piglets after normalization to total fungi (18 S) (Data were analyzed by two-tailed Student’s t-test, *n* = 10). **b** Hydrogen peroxide content in the colonic tissue; **c**, **d** SOD and GPX activity (*n* = 6, Data were analyzed with one-way ANOVA and presented as mean ± SEM); **e** *C. tropicalis* cells were cultured in yeast extract-peptone-dextrose medium. The cell density was adjusted to an OD_600_ of 1.0 and diluted to a 10^-5^ dilution by 10-fold serial dilution. The cells were incubated at 37 °C and treated with different levels of hydrogen peroxide, and then the growth of *C. tropicalis* was assessed. Data were analyzed with one-way ANOVA and presented as mean ± SEM, *n* = 3. *P < 0.05, **P < 0.01, ***P < 0.001 (*C. tropicalis* cells did not treat with hydrogen peroxide compared with cells treated with 1 mM or 2 mM hydrogen peroxide). **f** MPO activity in the colonic tissue; Pre-weaned piglets, samples were collected one day before weanling; Post-weaned C, samples were collected from healthy piglets three days after weanling; Post-weaned D, samples were collected from diarrheal piglets three days after weanling, *n* = 6. **g** Localization of neutrophil infiltration (MPO-positive, red) and NETs (Histone H2A, green) within the intestinal mucosa. Scale bar = 100 μm. **h** The MPO-DNA level in cell culture supernatant (*n* = 4). **i** *C. tropicalis* survival (*C. tropicalis* were co-cultured with hydrogen peroxide-treated neutrophils, *n* = 4). Data were analyzed with one-way ANOVA and presented as mean ± SEM. *P < 0.05, **P < 0.01.

We first confirmed that the relative abundance of *C. tropicalis* in the colonic mucosa decreased in diarrheal piglets (Fig. 3a). Weaned piglets are characterized by the excessive accumulation of ROS^14^. We confirmed that hydrogen peroxide content increased in the colonic tissue of weaned piglets (Fig. 3b). Notably, piglets with diarrhea showed remarkable changes. Superoxide dismutase (SOD) and glutathione peroxidase (GPX) activities were significantly decreased in post-weaned piglets with diarrhea (Fig. 3c, d). To explore whether the overaccumulation of hydrogen peroxide directly inhibits *C. tropicalis*, we cultured this species with different concentrations of hydrogen peroxide. We found that concentrations of 1 mM or higher inhibited the growth of *C. tropicalis* (Fig. 3e). However, a relatively low concentration of hydrogen peroxide (0.2 and 0.02 mM) had no inhibitory effect. As it did not reach such high concentrations of hydrogen peroxide (1 mM or higher) in the piglets under weaning stress (Supplementary Fig. 3), we then explored whether hydrogen peroxide indirectly affected *C. tropicalis* growth. It has been suggested that ROS are required for the formation of NETs from neutrophils, which can capture and kill *Candida* spp^15,16^. We first detected an increase in MPO activity in the colonic tissue of piglets with diarrhea, indicating the infiltration of polymorphonuclear neutrophils (Fig. 3f). We further observed increased protein expression of MPO and Histone H2A (Fig. 3g), which confirmed increased NET formation in the colon.

To explore whether NETs could kill *C. tropicalis*, we separated polymorphonuclear neutrophils from peripheral blood collected from weaned piglets and treated them with 0.02 mM hydrogen peroxide. We observed that hydrogen peroxide induced NET formation as did phorbol-12-myristate-13-acetate (PMA, a commonly known inducer of NETs), as indicated by an increase in the MPO–DNA complex ratio (Fig. 3h). We measured extracellular killing by first treating polymorphonuclear neutrophils with hydrogen peroxide and then cytochalasin D (cyt D) to block phagocytosis. The results suggested that hydrogen peroxide-induced NETs could kill *C. tropicalis*, as the survival ratio of *C. tropicalis* was found to be only approximately 70% (Fig. 3i). However, the addition of DNase-1, which dismantles the NET structure, reduces the killing of *C. tropicalis* by polymorphonuclear neutrophils. Taken together, these data suggest that NETs induced by ROS kill *C. tropicalis*.

### ROS promote NET release depending on dectin-1

**Fig. 4:**
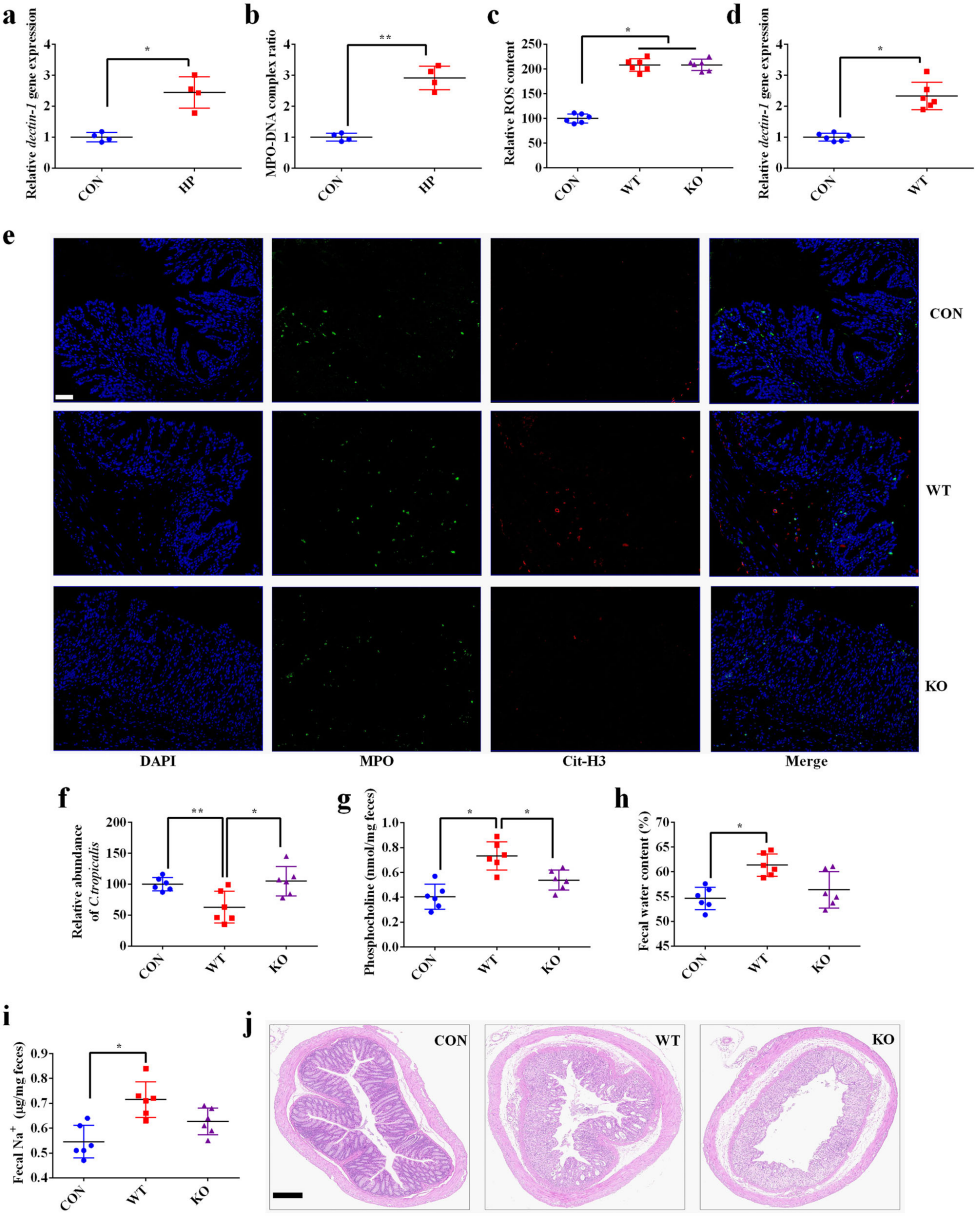
ROS promote NET release depending on dectin-1. Relative *dectin-1* expression **a** and MPO-DNA complex ratio **b** in neutrophils from wild-type mice after treated with 0.02 mM hydrogen peroxide; HP, hydrogen peroxide. Data were analyzed using a two-tailed Student’s t-test and are presented as the mean ± SEM. *n* = 4. *P < 0.05, **P < 0.01. Relative ROS content **c** and *dectin-1* gene expression **d** in the colonic tissue of mice; **e** Localization of neutrophil infiltration (MPO-positive, green) and NETs (Cit-H3, red) within the intestinal mucosa (Scale bar = 50 μm); **f** Relative abundance of *C. tropicalis* in the colonic mucosa*;* **g** Phosphocholine content in the feces; **h** Fecal water content; **i** Fecal Na^+^ content; **j** Colonic histomorphology (Scale bar = 200 μm). WT wild-type mice, KO dectin-1 knockout mice. Dectin-1 knockout and wild-type mice were instilled with 0.2 mL hydrogen peroxide solution in PBS (0.02 mM) via the intrarectal route once a day for 1 week. Data were analyzed using one-way analysis of variance (ANOVA) and presented as the mean ± SEM. *n* = 6. *P < 0.05, **P < 0.01.

It has been suggested that ROS production is critical for the activation of dectin-1 signaling^17^, which participates in NET release^18^. We found that the expression of *dectin-1* was significantly increased in the colonic tissue of diarrheal piglets (Supplementary Fig. 4), suggesting that ROS accumulation might activate dectin-1 in weaned piglets. To explore whether dectin-1 mediates the effects of ROS on NET release, we first isolated neutrophils from the peripheral blood of dectin-1-knockout and wild-type mice and treated them with hydrogen peroxide. We found increases in *dectin-1* expression and the MPO–DNA complex ratio in neutrophils from wild-type mice (Fig. 4a, b), whereas the MPO–DNA complex ratio was not changed in neutrophils from dectin-1-knockout mice (Supplementary Fig. 5). These results suggested that dectin-1 is critical for ROS-induced NET release. To further investigate whether the activation of dectin-1 was sufficient to promote NET release, we treated neutrophils from wild-type mice with β-D-glucan, an activator of dectin-1. We observed an increase in the MPO–DNA complex ratio in neutrophils (Supplementary Fig. 6a). Since the activation of dectin-1 signaling induces ROS production^17^, which was also observed in our results (Supplementary Fig. 6b), we further treated the cells with glutathione to eliminate ROS (Supplementary Fig. 6c). The results showed that the MPO–DNA complex ratio was not affected (Supplementary Fig. 6d). Consequently, our results further confirmed that dectin-1 activation is sufficient to induce NET release.

To further confirm the critical role of dectin-1 in hydrogen peroxide-induced diarrhea, we administered 0.02 mM hydrogen peroxide to dectin-1-knockout and wild-type mice through the rectum. We found that ROS content and *dectin-1* expression were increased in the colonic tissues of wild-type mice (Fig. 4c, d). Additionally, increased expression of MPO and Cit-H3 indicated increased NET formation (Fig. 4e). Furthermore, hydrogen peroxide decreased the abundance of *C. tropicalis* (Fig. 4f) and increased phosphocholine (Fig. 4g), water (Fig. 4h), and Na^+^ (Fig. 4i) content in the feces of wild-type mice. However, although ROS content increased (Fig. 4c) and colonic histomorphology was impaired in dectin-1-knockout mice (Fig. 4j), no other alterations, such as those in wild-type mice, were observed. These results further suggest that dectin-1 is essential for ROS-induced NET formation and diarrhea.

### Phosphocholine drives water efflux to promote the clearance of pathogenic bacteria

**Fig. 5:**
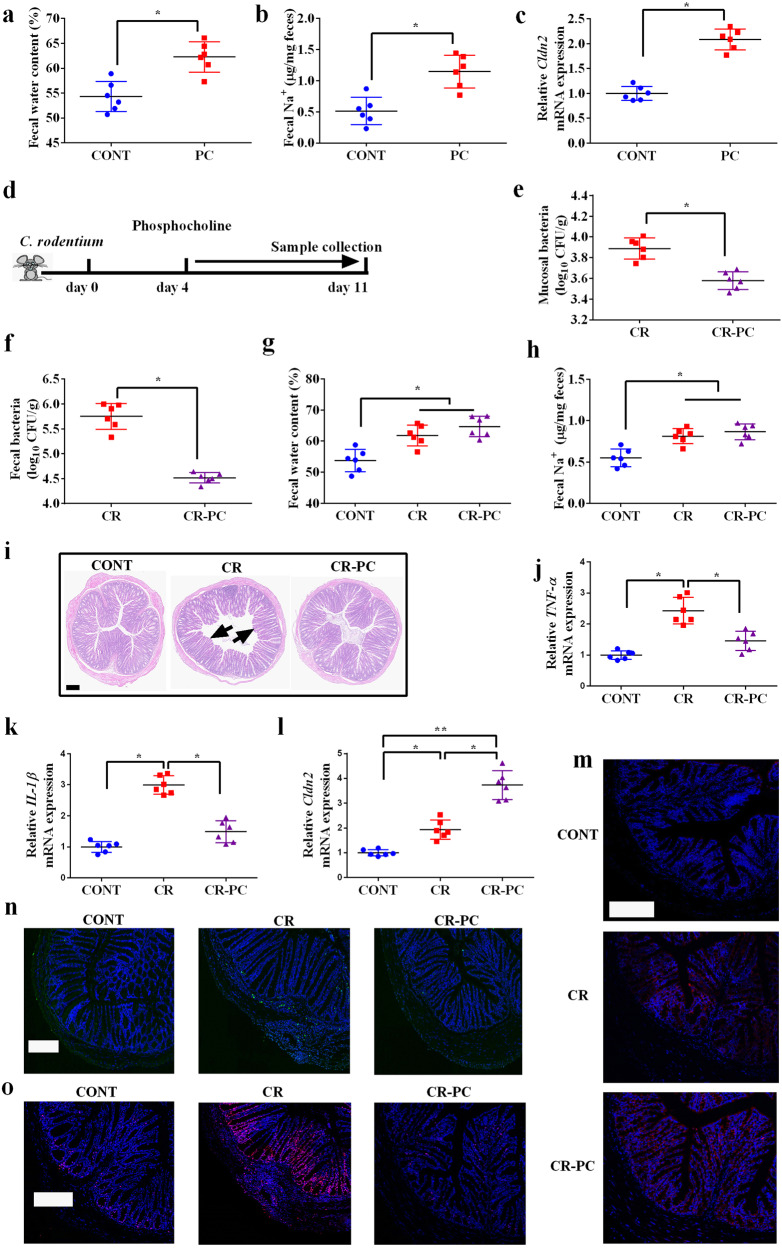
Phosphocholine drives water efflux to promote the clearance of pathogenic bacteria. **a** Fecal water content; **b** Fecal Na^+^ content; **c** Relative *Cldn2* mRNA expression; C57BL/6 male mice were either orally administrated with 0.1 mL PBS (CONT group) or 0.1 mL phosphocholine at a concentration of 2.5 mol/L (PC group) for 7 days. Data were analyzed by two-tailed Student’s t-test and presented as mean ± SEM. n = 6. *P < 0.05. **d** Schematic design; **e** Mucosal bacterial content; **f** Fecal bacterial content; **g** Fecal water content; **h** Fecal Na^+^ content; **i** Colonic morphology (Scale bar = 200 μm; arrows, impaired villus); Relative mRNA expression of *TNF-α* **j**, *IL-1β* **k**, and *Cldn2* **l**; Data were analyzed either with two-tailed Student’s t-test or one-way ANOVA. Data were presented as mean ± SEM. *n* = 6. *P < 0.05, **P < 0.01. **m** Claudin-2 expression (Blue, DAPI; Red, Claudin-2); **n** TUNEL staining (Blue, DAPI; Green, apoptotic cells); **o** Ki67 expression (Blue, DAPI; Red, Ki67). Scale bar = 200 μm. CONT, control mice; CR, mice were orally inoculated with 2 × 10^9^ CFU *C. rodentium* in a volume of 0.1 mL sterile PBS; CR-PC, mice were orally inoculated with *C. rodentium* and then 0.1 mL phosphocholine at a concentration of 2.5 mol/L was orally gavaged into mice once daily beginning on day 4 of *C. rodentium* infection.

To explore whether phosphocholine could induce diarrhea, mice were orally gavaged with phosphocholine. We found that phosphocholine treatment increased fecal water and Na^+^ content (Fig. 5a, b). Moreover, the expression of *Cldn2*, which has been shown to promote paracellular Na^+^ and water efflux^19^, was increased (Fig. 5c). However, phosphocholine treatment did not cause morphological damage (Supplementary Fig. 7a), increase the inflammatory response (*TNF-α* and *IL-1β* gene expression) (Supplementary Fig. 7b, c), or affect tight junction functions (*Tjp1* and *Ocln* gene expression) (Supplementary Fig. 7d, e). These results suggest that, although phosphocholine drives water efflux, it does not cause pathological changes in the intestine.

To further explore whether phosphocholine-induced water efflux protects against pathogen invasion, we first infected mice with *Citrobacter rodentium* and then treated them with phosphocholine (Fig. 5d). The results showed that *C. rodentium* numbers in both mucosa and feces were significantly reduced after phosphocholine treatment (Fig. 5e, f), confirming that phosphocholine promoted the clearance of pathogenic bacteria. Phosphocholine further increased fecal water and Na^+^ content in *C. rodentium*-infected mice; however, these changes were not significant (Fig. 5g, h). Additionally, phosphocholine protected mice from morphological damage (Fig. 5i) and inflammatory responses (*TNF-α* and *IL-1β* gene expression) (Fig. 5j, k) induced by *C. rodentium* infection, and increased *Cldn2* expression (Fig. 5l, m). Moreover, phosphocholine alleviated *C. rodentium*-induced apoptosis, as indicated by decreased caspase-3 expression (Fig. 5n), and epithelial proliferation, as indicated by decreased Ki67 expression (Fig. 5o). Collectively, these results indicated that phosphocholine drives water efflux to promote the clearance of pathogenic bacteria and maintain intestinal health.

### Phosphocholine decreases fluid absorption by increasing cAMP levels via activating adenylyl cyclase

**Fig. 6:**
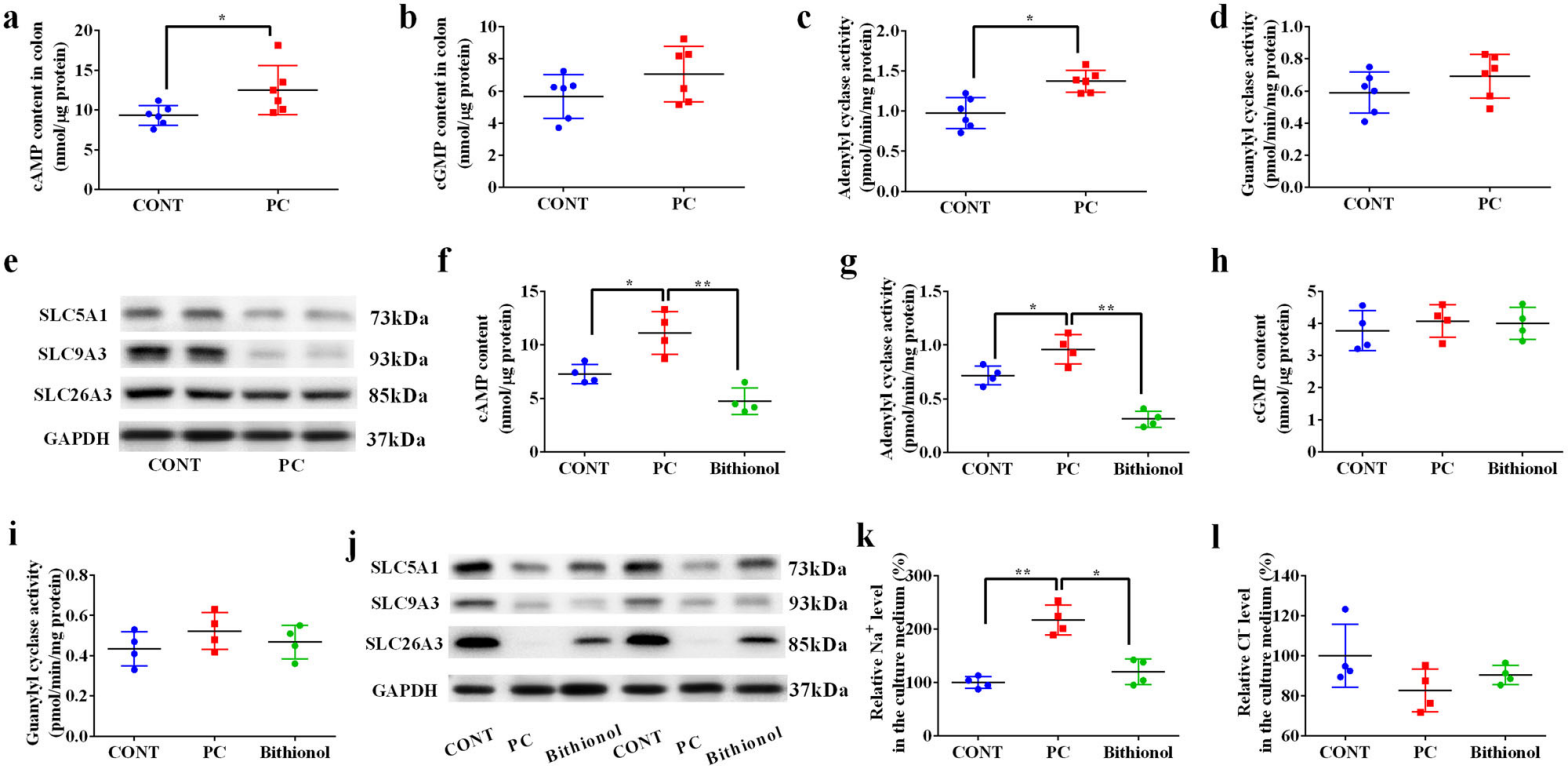
Phosphocholine decreases fluid absorption by increasing cAMP levels via the activation of adenylyl cyclase. The levels of cAMP **a** and cGMP **b** in the colon, and the activity of adenylyl cyclase **c** and guanylyl cyclase **d**; data were analyzed with two-tailed Student’s t-test and presented as mean ± SEM. *n* = 6. *P < 0.05. **e** Western blots showing the protein expression of SLC9A3, SLC5A1, SLC26A3, and GAPDH in the colon; cAMP content **f**, adenylyl cyclase activity **g**, cGMP content **h**, and guanylyl cyclase activity **i** in the primary colonocytes; **j** Western blots showing the protein expression of SLC9A3, SLC5A1, SLC26A3 and GAPDH in the primary colonocytes; Relative level of Na^+^ **k** and Cl^-^ **l** in the culture medium. Data were analyzed using one-way analysis of variance (ANOVA) and presented as the mean ± SEM. *n* = 4. *P < 0.05, **P < 0.01.

Next, we investigated the mechanism by which phosphocholine affected Na^+^ and water efflux. Ca^2+^ and cyclic nucleotides, which serve as intracellular messengers, have been suggested to play critical roles in regulating the activity of Na^+^ transporters^20^. We found that the level of cAMP was significantly increased (Fig. 6a), whereas no change was observed in the levels of cGMP (Fig. 6b) and Ca^2+^ (Supplementary Fig. 8) in the colonic tissues of mice administered phosphocholine. Consistently, phosphocholine administration increased adenylyl cyclase activity (Fig. 6c), but did not affect guanylyl cyclase activity (Fig. 6d) in the colon. Active Na^+^ transporters across the epithelium mainly include sodium/hydrogen exchanger 3 (SLC9A3), sodium/glucose cotransporter 1 (SLC5A1), and Cl^−^/HCO3^−^ exchanger (SLC26A3). Our data showed that the expression of all these Na^+^ transporters decreased (Fig. 6e) following phosphocholine administration. These results indicate that phosphocholine decreases fluid absorption by increasing the cAMP levels.

To further explore whether adenylyl cyclase critically mediates the effects of phosphocholine, we isolated primary colonocytes and treated them with the adenylyl cyclase inhibitor, bithionol. We found that phosphocholine increased cAMP levels and adenylyl cyclase activity in primary colonocytes, whereas bithionol reversed these effects (Fig. 6f, g). Phosphocholine and bithionol had no effect on cGMP levels or guanylyl cyclase activity (Fig. 6h, i). Meanwhile, bithionol reversed the inhibitory effects of phosphocholine on the protein expression of SLC26A3 and SLC5A1 but did not affect SLC9A3 expression (Fig. 6j). In addition to alterations in Na^+^ transporters, phosphocholine increased Na^+^ levels in the culture medium, whereas bithionol reversed this change (Fig. 6k). The Cl^−^ level was also decreased by phosphocholine and reversed by bithionol; however, these changes were not statistically significant (Fig. 6l). In conclusion, these data suggest that adenylyl cyclase critically mediates the effect of phosphocholine on fluid absorption.

## Discussion

Gut microbiota dysbiosis has been proven to be critically involved in the occurrence and development of diarrhea. Previous studies have focused on alterations in the bacterial composition and their metabolites, which can induce diarrhea primarily by affecting intestinal fluid movement and inflammatory responses^6,9,21^. Our data further suggest that changes in intestinal fungi, their metabolism, and host responses to these alterations could contribute to the development of diarrhea. Interestingly, we found a significant difference in intestinal *C. tropicalis* abundance between diarrheal piglets and healthy control piglets. It is commonly considered that *C. tropicalis* is emerging as a prevalent pathogenic yeast^22^. As one of the most abundant species of indigenous fungi that colonizes the gastrointestinal tracts^23^, *C. tropicalis* can penetrate mucosal layers and cause infection in immunocompromised individuals^22^. Unexpectedly, we observed a decrease in the abundance of *C. tropicalis* in piglets with diarrhea. These decreases were negatively correlated with increased levels of phosphocholine, which has been shown to drive water efflux and induce diarrhea. Although limited evidence supports the hypothesis that diarrhea could be beneficial, we and others have found that the initial stage of secretory diarrhea could help maintain intestinal function and promote pathogen clearance^19^. Previous studies have reported that *Candida* spp., including *C. tropicalis* in stool, are associated with inflammatory bowel diseases^10,24^, although this is still controversial. However, our results suggest that *C. tropicalis* colonization of the intestine helps maintain the balance of intestinal metabolites.

Weaning is a stressful event that causes the overproduction of ROS, which results in inhibition of the antioxidant system and gastrointestinal disorders, leading to diarrhea^14^. Our data confirmed that post-weaned piglets with diarrhea had a significantly increased ROS content and decreased antioxidant enzyme activity, whereas healthy post-weaned piglets showed no such changes. It is possible that piglets without diarrhea have a well-developed antioxidant system that eliminates ROS; however, piglets with low antioxidant capacities cannot deal with the overproduction of ROS, which kills *C. tropicalis* and induces diarrhea. It has been suggested that ROS, acting as secretagogues, may contribute to diarrhea^25^. Unlike piglets, which had acute diarrhea immediately after weaning, wild-type mice showed signs of diarrhea after rectal administration of hydrogen peroxide for almost 1 week. Thus, it is possible that, although ROS are the major cause of diarrhea, such processes could be mediated by many pathways along the entire intestine, including inflammatory responses and mucin secretion, in weaned piglets^9^. Nevertheless, using dectin-1-knockout mice, our data indicated that ROS-induced diarrhea in the colon was dependent on dectin-1. Specifically, by targeting dectin-1, ROS promotes the release of NETs, which kill *C. tropicalis*. The decreased consumption of phosphocholine by *C. tropicalis* was responsible for its accumulation, which further induced diarrhea. Of note, although we found that *C. tropicalis* could consume phosphocholine for its growth in vitro, the specific underlying mechanisms have not been elucidated, and additional experiments are required.

It is commonly considered that diarrhea is closely associated with intestinal inflammation and epithelial impairment^20^. However, a recent study reported that the overexpression of claudin-2 or osmotic agents could promote water efflux, wash away adherent pathogens, and limit the development of diseases as a response to the innate defense against invasive pathogens^19^. Interestingly, our data further indicate that phosphocholine has effects similar to those of osmotic agents. Importantly, phosphocholine promoted claudin-2 expression in the colon, suggesting that it may activate innate defense mechanisms. Although phosphocholine decreased the fecal and mucosal abundance of *C. rodentium* and alleviated morphological damage and inflammation, it did not further increase the fecal water and Na^+^ content. This could be because phosphocholine increased paracellular permeability via the pore and leak pathways while improving the unrestricted pathway. Although phosphocholine is involved in intracellular signal transduction, the specific mechanisms by which phosphocholine regulates claudin-2 expression require further exploration. Water flux can occur through paracellular, transcellular, and transport-related processes. Since most studies have failed to prove the effects of aquaporins on intestinal water transport^26,27^, we did not observe their alterations in response to phosphocholine. However, we confirmed that phosphocholine could increase the expression of Na^+^ transporters by enhancing cAMP content by targeting adenylyl cyclase in uninfected mice and in vitro in primary colonocytes. Phosphocholine can be synthesized via the phosphorylation of choline, using ATP as a phosphate donor^28,29^. It is possible that the over-accumulation of phosphocholine comprised an end-product that inhibited the activity of choline kinase and the consumption of ATP, which might have indirectly promoted the formation of cAMP. Phosphocholine levels rapidly increased in post-weaned piglets and immediately induced diarrhea. However, when phosphocholine was administered to mice, it took much longer to induce diarrhea. This might be because a relatively high abundance of *C. tropicalis* colonizing the intestine, as previously discussed, consumed phosphocholine^23^. Additionally, although we proved that phosphocholine alone could induce diarrhea, many other processes may be involved in the occurrence of diarrhea in post-weaned piglets^6,8,9^.

In conclusion, our results suggest that post-weaned animals with diarrhea exhibit alterations in their fecal mycobiota, which may be involved in the occurrence of diarrhea. Specifically, the overaccumulation of ROS caused by weaning stress promotes the formation of NETs, which is dependent on dectin-1, resulting in *C. tropicalis* killing. A decrease in *C. tropicalis* results in less consumption of phosphocholine, leading to its accumulation in the colon, followed by phosphocholine-driven water efflux via a decrease in fluid absorption mediated by the activation of adenylyl cyclase, which promotes the clearance of pathogenic bacteria. Collectively, our data indicate that disruption of the balance between colonic fungi and consumption of intestinal metabolites might contribute to diarrhea and pathogen clearance. Additionally, we elucidated the mechanisms by which weaning stress leads to diarrhea, indicating that diarrhea in the early stages could be a beneficial response to pathogen invasion.

## Methods

### Ethics statement

All mice were housed under specific pathogen-free conditions) and piglets were cared for at the Institute of Subtropical Agriculture, Chinese Academy of Sciences. Animal procedures were approved by the Protocol Management and Review Committee of the Institute of Subtropical Agriculture, Chinese Academy of Sciences (ISA2021091).

### Animals

Crossbred piglets (Duroc × Landrace × Yorkshire), pre-weaned or weaned at the age of 21 days were obtained from Tianxin Pig Industry (Changsha, China). At the end of the experiments, piglets were humanely anaesthetized with 50 mg sodium pentobarbital/kg body weight via intravenous injection and bled by exsanguination to reduce suffering for sample collection. Dectin-1 knockout mice with a C57BL/6 background and wild-type littermates aged 8–10 weeks were purchased from Cyagen (Guangzhou, China).

### Selection of diarrheal piglets

Fifty litters of piglets (Duroc × Landrace × Yorkshire) weaned at the age of 21 days from a commercial farm (Hunan, China) were selected. From days 1 to 3 post-weaning, thirty-eight diarrheal piglets (18 males and 20 females) were chosen, and samples were collected immediately when diarrhea occurred. Twenty-seven healthy piglets (13 males and 14 females) were chosen as controls. Those piglets with loose, semi-liquid or watery feces were designated as diarrhea, while those piglets with hard or normal consistency feces were designated as controls^6^. During the experiment, all piglets were fed a diet based on the nutrient requirements of the NRC2012 and given free access to feed and water.

### Fungal ITS gene high-throughput sequencing and data analysis

Fresh fecal samples were collected and total microbial genomic DNA was extracted using the QIAampDNA stoolMiniKit (Qiagen, Shanghai, China)^30^. Fungal ITS1/ITS2 regions were amplified using the following primers: ITS1F 5′-CTTGGTCATTTAGAGGAAGTAA-3′ and ITS2R 5′-GCTGCGTTCTTCATCGATGC-3′. The PCR was performed at an annealing temperature of 50 °C for 30 cycles. Sequencing libraries were generated using the TruSeq® DNA PCR-Free Sample Preparation Kit (Illumina, Shanghai, China) and sequenced on the Illumina MiSeq platform using Novogene (Beijing, China). Sequences were analyzed using the Uparse software, and OTUs were clustered with 97% sequence similarity. Alpha and beta diversities were calculated using QIIME (V1.9.0) and R software (V2.15.3). Principal coordinates analysis (PCoA) was performed using the WGCNA, ggplot2, and stat packages in R software (V2.15.3).

### Untargeted metabolomics

Fecal samples were mixed with sterile water, supernatants were collected after centrifugation, and liquid chromatography-mass spectrometry (LC-MS)-based metabolomics was performed by Novogene. The LC-MS platform was equipped with a Vanquish UHPLC and Thermo Scientific Q-Exactive high-resolution mass spectrometer. The metabolites were identified using the MS/MS database. The acquired data were imported for SIMCA Statistical Analysis and principal component analysis (PCA). The differential metabolites were further analyzed by the variable importance in the projection and *P* values from two-tailed Student’s t-test.

### Candida tropicalis cultivation

*C. tropicalis* cells (ATCC 20026) were cultured in a yeast extract-peptone-dextrose medium. The cell density was adjusted to an OD_600_ of 1.0, and diluted to a 10^−5^ dilution by 10-fold serial dilution. Cells were incubated at 37 °C to assess their growth. The treatments are described in the methods section.

### Determination of phosphocholine and choline contents

Phosphocholine and choline concentrations in the colonic tissue and culture medium were assayed. Colonic tissue was homogenized in methanol and centrifuged at 4 °C for 8 min at 16,000 *g*. The supernatant was then evaporated and dissolved in water. Finally, all the solutions, including the culture medium, were filtered through a polysulfone filter. To assay choline concentration, 1 mL sample was incubated at 37 °C for 10 min in a total volume of 2 mL mixture with 100 mM Tris-HCl, 1 U/ml peroxidase, 1 U/ml choline oxidase, 0.15 mM 4-aminoantipyrine, 1.5 mM phenol, and 0.5% Triton X-100. The mixture was then measured at a wavelength of 500 nm. To assay the choline plus phosphocholine content, 2 U/ml alkaline phosphatase was added to the mixture. The phosphocholine content was calculated by subtracting the choline content from the content of choline and phosphocholine^31^.

### DNA isolation and Real Time qPCR for fungi quantification

*Candida tropicalis* was qualified by normalizing to total fungi^32^. DNA was isolated from colonic mucosa following lyticase treatment and processing using a QIAmp DNA mini kit (Qiagen, Shanghai, China). RT-qPCR was performed using the SYBR Green PCR kit (Thermo Fisher, Shanghai, China) according to the manufacturer’s instructions. Primer sequences are listed in Supplementary Table 1.

### Determination of hydrogen peroxide in the colon

The distal colon was collected, and hydrogen peroxide was determined using a commercially available kit (BYabscience, Nanjing, China), according to the manufacturer’s instructions.

### Determination of myeloperoxidase activity in the colon

Colonic tissue was homogenized in 10% (w/v) ice-cold PBS containing 0.5% hexadecyl-trimethyl ammonium hydroxide. The supernatant was collected after centrifugation at 1000 *g* at 4 °C for 10 min, and then treated with PBS, 0.0005% hydrogen peroxide and 0.17 mg/mL 3,3′-dimethoxybenzidine to a total volume of 50 μL. Finally, MPO activity was analyzed by determining the hydrogen peroxide-dependent oxidation of 3,3′-dimethoxybenzidine^33^.

### Immunofluorescence staining

The distal colons were fixed with 4% formaldehyde and embedded in paraffin. After being cut into 8-μm sections, the samples were incubated with primary antibodies, including MPO (Abcam Cat#ab208670; RRID: AB_2864724; Dilution, 1:200), Histone H2A (Abcam Cat#ab177863; RRID: not found; Dilution, 1:400), Cit-H3 (Cell Signaling Technology Cat#97272; RRID, not found; Dilution, 1:800), Claudin-2 (Abcam, Cat#ab53032; RRID:AB_869174; Dilution, 1:200), and Ki67 (Abcam Cat#ab15580, RRID:AB_443209; Dilution, 1:100) at 4 °C overnight and then incubated with secondary antibodies. Finally, the sections were mounted on a slide with 4′6′-diamidino-2-phenylindole (DAPI) reagent, and representative pictures were obtained under a fluorescent microscope.

### Neutrophils isolation

Peripheral blood collected from either weaned piglets or mice was mixed with Optiprep (Axis-Shield, Shanghai, China) and centrifuged at 800 *g* for 30 min. Neutrophils in the lower interphase were obtained and washed with PBS. After treatment with red blood cell lysis buffer, the neutrophils were resuspended in RPMI 1640 medium containing 1% HEPES.

### Quantification of MPO-DNA

After re-suspended at 2 × 10^6^ mL^-1^, porcine neutrophils were treated either with 20 nM PMA or 0.02 mM hydrogen peroxide for 2 h. To quantify NETs in cell culture supernatant, a capture ELISA kit for MPO (Hycult biotech, Wayne, PA, USA) and a peroxidase-labeled anti-DNA antibody (Roche, Shanghai, China) were used according to the manufacturers’ protocol, respectively.

### In vitro C. tropicalis killing assay

Porcine neutrophils were incubated for 2 h at 37 °C in RPMI medium with 0.02 mM hydrogen peroxide after resuspension at 2 × 10^6^ mL^−1^. Next, 10 μg/mL cyt D was added to the medium (Sigma-Aldrich, Shanghai, China) for 20 min before infection with *C. tropicalis*. The moi was 0.01 (*C. tropicalis*: neutrophils). Before infection with neutrophils, *C. tropicalis* was sub-cultured in YPD at 30 °C to induce yeast-forming cells. To degrade NETs, neutrophils were preincubated with 100 U/mL RNase and protease-free DNase-1. *C. tropicalis* survival was determined as the percentage of *C. tropicalis* colonies after incubation with neutrophils compared with those incubated alone^15^.

### Hydrogen peroxide administration through the rectum

Dectin-1-knockout mice on a C57BL/6 background were purchased from Cyagen (Guangzhou, China). Wild-type and dectin-1 knockout mice were instilled with 0.2 mL PBS or 0.2 mL hydrogen peroxide solution in PBS (0.02 mM) via the intrarectal route once a day for 1 week.

### Fecal water and Na^+^ determination

Fresh feces were collected and immediately weighed. The samples were then incubated in a dry oven at 60 °C for 24 h and weighed again. Fecal water was calculated as the fraction of the total mass lost. Dried feces were rehydrated with deionized water (5× the initial water volume). The solution was then homogenized and centrifuged to remove insoluble debris. Finally, the fecal Na^+^ content was determined using a Compact Sodium Ion Meter (LAQUAtwin B-743, Horiba, Japan). Stool Na^+^ mass was normalized to dry stool mass.

### RT-qPCR analysis

Total RNA was extracted using TRIzol reagent (Invitrogen, Shanghai, China) and cDNA was obtained using the Reverse Transcription Reagent Kit (Takara, Dalian, China). RT-qPCR was performed using the SYBR Green PCR Kit (Thermo Fisher Scientific) according to the manufacturer’s instructions. Primer sequences used are listed in Supplementary Table 1.

### Determination of ROS content

Colonic tissues were obtained, digested, and filtered to prepare cellular suspensions. The ROS content was determined using a kit (BYabscience) according to the manufacturer’s instructions.

### Morphology observation

The distal colon was fixed with 4% formaldehyde and the sample was sliced into 8-μm sections. Hematoxylin and eosin (HE) staining was performed to observe morphological changes.

### Phosphocholine administration

Mice were orally administrated with 0.1 mL phosphocholine (MCE, Shanghai, China) was orally administered to the mice at a concentration of 2.5 mol/L for 7 days.

### Citrobacter rodentium infection

*C. rodentium* strain DBS100 (ATCC 51459) was grown overnight in LB broth at 37 °C. After fasting for 4 h, the mice were orally inoculated with 2 × 10^9^ CFU *C. rodentium* in a volume of 0.1 mL sterile PBS. To determine the role of phosphocholine in infection, 0.1 mL phosphocholine at a concentration of 2.5 mol/L was orally administered to mice once daily, beginning on day 4 of *C. rodentium* infection.

### Citrobacter rodentium quantification

Fresh stool samples were collected and homogenized in sterile PBS. Colons were flushed with ice-cold sterile PBS, and the distal colon was collected, weighed, and homogenized to determine the presence of mucosa-associated bacteria. Colonic and fecal homogenates were serially diluted and plated onto MacConkey agar. After growth at 37 °C for 20 h, pink colonies were identified as *C. rodentium*.

### Assessment of apoptosis

The distal colon was fixed with 4% formaldehyde, and the sample was sliced into 5-μm section. TUNEL staining was performed to determine apoptosis using an in situ cell death detection kit (Roche, Basel, Switzerland). Sections were then mounted on a slide with DAPI, and representative images were obtained under a fluorescent microscope.

### Isolation of colonic epithelium

The colon was firstly dissected and flushed four times with ice-cold PBS to isolate colonic epithelium^34^. Then, the colon was inverted by inserting a gavage needle and both ends of the inverted colon were secured with sutures. The inverted colon was submerged in ice-cold Cell Recovery solution (Corning, Shanghai, China). After deflation and re-inflation with the plunger of the syringe attached to the needle, an epithelial layer was obtained by dipping the inverted colon in cold PBS.

### Determination of Ca^2+^, cAMP and cGMP contents

The Ca^2+^ in the colonic tissue was determined using a kit (Sigma-Aldrich), according to the manufacturer’s instructions. The levels of cAMP and cGMP in the colonic tissue and primary colonocytes were measured using a cAMP and cGMP ELISA Kit (Meimian, Jiangsu Yutong, Nanjing, China) according to the manufacturer’s instructions. The protein levels were measured using a BCA protein quantification kit (Millipore, Shanghai, China). Ca^2+^, cAMP, and cGMP levels were normalized to protein concentration.

### Determination of cyclase activity

Adenylyl cyclase activity was measured firstly by mixing 30 μL of colonocytes (5 × 10^7^/mL) or colonic tissue homogenates (0.1 g/mL) to 10 μL of adenylyl cyclase activity assay buffer^35^. The reaction was terminated by the addition of 40 μL of 2% w/v sodium dodecyl sulfate. After added 5000 cpm of [^3^H]cAMP, the sample was diluted to 1 mL with dH_2_O and then eluted from Dowex column to alumina columns. Finally, cAMP was eluted by the addition of 4 mL of 0.1 M imidazole-HCl. To measure guanylyl cyclase activity, 15 μL of colonocytes (5 × 10^7^/mL) or colonic tissue homogenates (0.1 g/mL) were reacted with 10 μL of the K-gluc buffer^36^. The reaction was terminated by the addition of 40 μL of 0.4 M HCl. Then, 150 mg of Alumina N was added to 40 μL of the mixture and the reaction suspended in 0.5 mL of a Tris buffer. Finally, the solution was centrifuged for 15 min at 20,000 *g* and the supernatant (40 μL) was well mixed with a liquid scintillation mixture (4 mL). A liquid scintillation counter was used to count ^3^H and ^32^P radioactivity for the determination of adenylyl cyclase and guanylyl cyclase activity. Protein levels were measured using a BCA protein quantification kit (Millipore). The activities of adenylyl cyclase and guanylyl cyclase were normalized to protein concentration.

### Western blotting assay

Total protein was extracted and western blotting was performed to detect expression of target proteins^37^. A total of 30 μg of protein in each lane was separated using SDS-PAGE and subsequently blotted onto nitrocellulose membranes. The membranes were blocked with 5% skim milk and then incubated with antibodies against SLC9A3 (Bioss Cat#bs-8601R; RRID: AB_2928052; Dilution, 1:500), SLC5A1 (Bioss, Cat#bs-1128R; RRID: AB_10856441; Dilution, 1:500), SLC26A3 (Boster, Cat#A03335; RRID: not found; Dilution, 1:1000), and GAPDH (Abcam Cat# ab8245; RRID: AB_2107448; Dilution, 1:2000) overnight and then with secondary antibodies for 2 h at 22 ± 2 °C. The results were observed using the EZ-ECL reagent (Biological Industries, Shanghai, China). All blots were processed in parallel and derive from the same experiments. Original blots are provided in Supplementary Information.

### Determination of Cl^-^ content

The Cl^-^ was determined using a kit (Sigma-Aldrich), according to the manufacturer’s instructions.

### Statistics analysis

All data were analyzed using one-way ANOVA followed by the S-N-K post hoc test or two-tailed Student’s t-test, using SPSS Statistics software (version 18.0) and GraphPad Prism software (version 8.0). The results are expressed as the mean ± SEM, and *P* < 0.05 was considered significant.

### Reporting summary

Further information on research design is available in the Nature Research Reporting Summary linked to this article.

### Supplementary information

SUPPLEMENTAL MATERIAL

Reporting Summary

## Data Availability

The ITS gene sequence data were deposited in the NCBI SRA database (https://www.ncbi.nlm.nih.gov/sra/) under accession number PRJNA891366. All data needed to evaluate the conclusions in the paper are presented in the paper and/or Supplementary Materials. Additional data related to this study may be requested from the corresponding author.

## References

[1] Surawicz CM (2010). Mechanisms of diarrhea. Curr. Gastroenterol. Rep..

[2] GBD 2019 Under-5 Mortality Collaborators. (2021). Global, regional, and national progress towards Sustainable Development Goal 3.2 for neonatal and child health: all-cause and cause-specific mortality findings from the Global Burden of Disease Study 2019. Lancet..

[3] Li M (2012). An atlas of DNA methylomes in porcine adipose and muscle tissues. Nat. Commun..

[4] Lunney JK (2021). Importance of the pig as a human biomedical model. Sci. Transl. Med..

[5] Zhang Q, Widmer G, Tzipori S (2013). A pig model of the human gastrointestinal tract. Gut Microbes.

[6] Zhou X (2022). Intestinal accumulation of microbiota-produced succinate caused by loss of microRNAs leads to diarrhea in weanling piglets. Gut Microbes.

[7] Carroll IM, Ringel-Kulka T, Siddle JP, Ringel Y (2012). Alterations in composition and diversity of the intestinal microbiota in patients with diarrhea-predominant irritable bowel syndrome. Neurogastroenterol. Motil..

[8] Ren W (2022). Lower abundance of Bacteroides and metabolic dysfunction are highly associated with the post-weaning diarrhea in piglets. Sci. China Life Sci..

[9] Xia B (2022). Mucin O-glycan-microbiota axis orchestrates gut homeostasis in a diarrheal pig model. Microbiome..

[10] Liguori G (2016). Fungal dysbiosis in mucosa-associated microbiota of Crohn’s disease patients. J. Crohns. Colitis..

[11] Hoarau G (2016). Bacteriome and mycobiome interactions underscore microbial dysbiosis in familial Crohn’s disease. mBio.

[12] Chehoud C (2015). Fungal signature in the gut microbiota of pediatric patients with inflammatory Bowel disease. Inflamm. Bowel. Dis..

[13] Limon JJ (2019). Malassezia is associated with Crohn’s disease and exacerbates colitis in mouse models. Cell. Host. Microbe..

[14] Zhu LH, Zhao KL, Chen XL, Xu JX (2012). Impact of weaning and an antioxidant blend on intestinal barrier function and antioxidant status in pigs. J. Anim. Sci..

[15] Urban CF, Reichard U, Brinkmann V, Zychlinsky A (2006). Neutrophil extracellular traps capture and kill Candida albicans yeast and hyphal forms. Cell. Microbiol..

[16] Drummond RA, Brown GD (2011). The role of Dectin-1 in the host defence against fungal infections. Curr. Opin. Microbiol..

[17] Ma J, Becker C, Lowell CA, Underhill DM (2012). Dectin-1-triggered recruitment of light chain 3 protein to phagosomes facilitates major histocompatibility complex class II presentation of fungal-derived antigens. J. Biol. Chem..

[18] Bachiega TF (2016). Participation of dectin-1 receptor on NETs release against Paracoccidioides brasiliensis: Role on extracellular killing. Immunobiology..

[19] Tsai PY (2017). IL-22 upregulates epithelial claudin-2 to drive diarrhea and enteric pathogen clearance. Cell. Host. Microbe..

[20] Thiagarajah JR, Donowitz M, Verkman AS (2015). Secretory diarrhoea: mechanisms and emerging therapies. Nat. Rev. Gastroenterol. Hepatol..

[21] Hu J (2018). A microbiota-derived bacteriocin targets the host to confer diarrhea resistance in early-weaned piglets. Cell. Host. Microbe..

[22] Kothavade RJ, Kura MM, Valand AG, Panthaki MH (2010). Candida tropicalis: its prevalence, pathogenicity and increasing resistance to fluconazole. J. Med. Microbiol..

[23] Li J (2018). Fungi in gastrointestinal tracts of human and mice: from community to functions. Microb. Ecol..

[24] Sokol H (2017). Fungal microbiota dysbiosis in IBD. Gut..

[25] Gaginella TS, Kachur JF, Tamai H, Keshavarzian A (1995). Reactive oxygen and nitrogen metabolites as mediators of secretory diarrhea. Gastroenterology..

[26] Thiagarajah JR (2017). Aquaporin-3 mediates hydrogen peroxide-dependent responses to environmental stress in colonic epithelia. Proc. Natl Acad. Sci. USA.

[27] Yang B, Song Y, Zhao D, Verkman AS (2005). Phenotype analysis of aquaporin-8 null mice. Am. J. Physiol. Cell. Physiol..

[28] Kent C (1990). Regulation of phosphatidylcholine biosynthesis. Prog. Lipid. Res..

[29] Vance DE (1990). Boehringer Mannheim Award lecture. Phosphatidylcholine metabolism: masochistic enzymology, metabolic regulation, and lipoprotein assembly. Biochem. Cell. Biol..

[30] He L (2022). Fecal miR-142a-3p from dextran sulfate sodium-challenge recovered mice prevents colitis by promoting the growth of Lactobacillus reuteri. Mol. Ther..

[31] Nakagami K (1999). Increased choline kinase activity and elevated phosphocholine levels in human colon cancer. Jpn J. Cancer. Res..

[32] Leonardi I (2018). CX3CR1(+) mononuclear phagocytes control immunity to intestinal fungi. Science..

[33] Yuan D (2017). The evaluation of antioxidant and anti-inflammatory effects of eucommia ulmoides flavones using diquat-challenged piglet models. Oxid. Med. Cell. Longev..

[34] Nik AM, Carlsson P (2013). Separation of intact intestinal epithelium from mesenchyme. Biotechniques..

[35] Miller RA (2013). Biguanides suppress hepatic glucagon signalling by decreasing production of cyclic AMP. Nature..

[36] Takemoto N, Tachibanaki S, Kawamura S (2009). High cGMP synthetic activity in carp cones. Proc. Natl Acad. Sci. USA.

[37] Zhou X (2017). Serine alleviates oxidative stress via supporting glutathione synthesis and methionine cycle in mice. Mol. Nutr. Food Res..

