# Supplementary material for: Colonic phosphocholine is correlated with Candida tropicalis and promotes diarrhea and pathogen clearance
Source: NPJ Biofilms Microbiomes. 2023 Sep 4;9:62. doi: 10.1038/s41522-023-00433-0 (PMC10477305; doi:10.1038/s41522-023-00433-0)

Supplementary Table 1 Primers for quantification of gene expression by qPCR

	5'-3' Primer sequence
Fungi (18S)	F: ATTGGAGGGCAAGTCTGGTG<br>R: CCGATCCCTAGTCGGCATAG
C. tropicalis	F: TTTGGTGGCGGGAGCAATCCT<br>R: CGATGCGAGAACCAAGAGATCCGT
Porcine *Dectin-1*	F: TGGTCTCCGAGAAAGGAGTTC<br>R: ATGGAGCCATCCTCCCAAAG
Porcine *β-actin*	F: CTGCGGCATCCACGAAACT<br>R: AGGGCCGTGATCTCCTTCTG
Mouse *Dectin-1*	F: GGGTGCCCTAGGAGGTTTTT<br>R: AACCATGGCCCTTCACTCTG
Mouse *Cldn-2*	F: GCTCCGTGAGTATCTGGTCG<br>R: AAGGCCTAGGATGTAGCCCA
Mouse *TNF-α*	F: ATGAGAAGTTCCCAAATGGC<br>R: CTCCACTTGGTGGTTTGCTA
Mouse *IL-1β*	F: TGCCACCTTTTGACAGTGATG<br>R: AAGGTCCACGGGAAAGACAC
Mouse *Tjp1*	F: GAGCAGGCTTTGGAGGAGAC<br>R: TGGGACAAAAGTCCGGGAAG
Mouse *Ocln*	F: TTTCCTTAGGCGACAGCGG<br>R: GACATGCATCTCTCCGCCAT
Mouse *β-actin*	F: TGTCCACCTTCCAGCAGATGT<br>R: AGCTCAGTAACAGTCCGCCTAGA

Supplemental Figure 1 Effects of choline addition on *C. tropicalis* growth

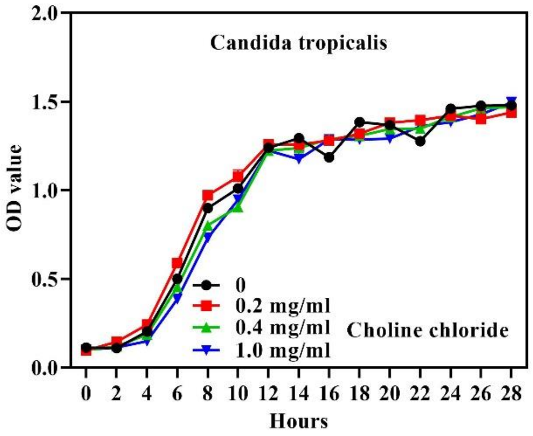

Supplemental Figure 2 Choline kinase activity and phosphocholine content in the colonic tissue

(a) Choline kinase activity; (b) Phosphocholine content. Data were analyzed with one-way ANOVA and presented as mean ± SEM, n=10. Diarrhea, diarrheal piglets; Control, healthy piglets without diarrhea.

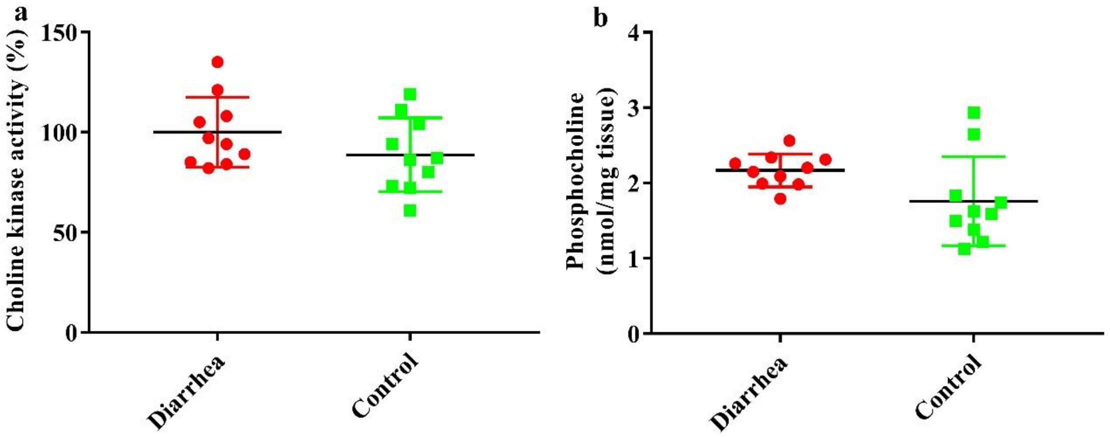

Supplemental Figure 3 The hydrogen peroxide content in the serum of piglets before and after weanling

Data were analyzed with one-way ANOVA and presented as mean ± SEM, n=6. Pre-weaned piglets, samples were collected one day before weanling; Post-weaned C, samples were collected from healthy piglets three days after weanling; Post-weaned D, samples were collected from diarrheal piglets three days after weanling.

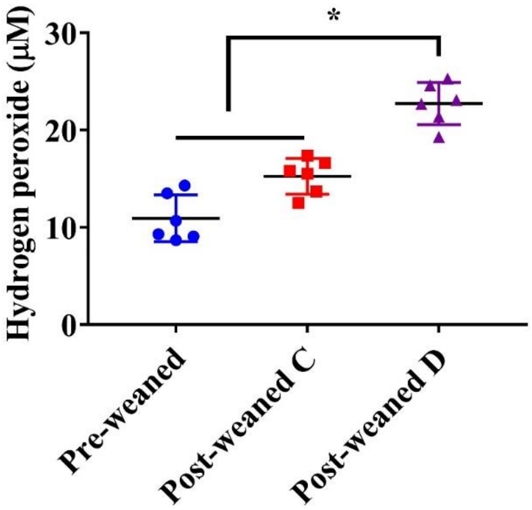

Supplemental Figure 4 ***Dectin-1*** **expression in the piglets before and after weanling**

Data were analyzed with one-way ANOVA and presented as mean ± SEM, n=6. *P <0.05.

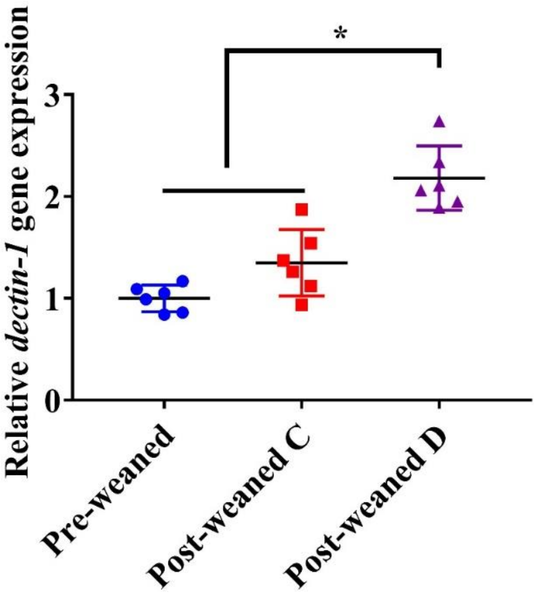

Supplemental Figure 5 MPO-DNA complex ratio in neutrophils collected from dectin-1-knockout mice and treated with hydrogen peroxide

CON, control neutrophils; HP, neutrophils treated with 0.02 mM hydrogen peroxide. Data were analyzed with two-tailed Student's t-test and presented as mean ± SEM, n=4.

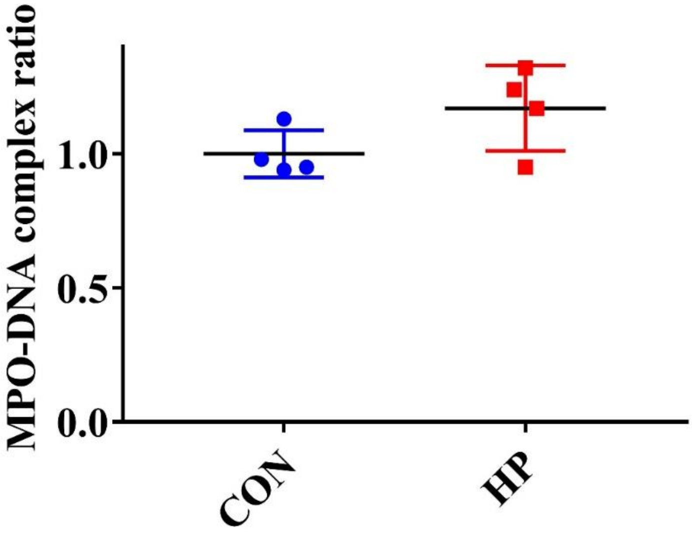

Supplemental Figure 6 Dectin-1 activation promotes NETs release in neutrophils

Neutrophils were treated with 50 μg/mL β-D-Glucan for 24 h. (a) MPO-DNA complex ratio; (b) Relative ROS content; (c) Relative ROS content; (d) MPO-DNA complex ratio. Neutrophils were treated with 50 μg/mL β-D-Glucan for 24 h and then with 2 mM GSH for another 24 h. Data were analyzed by two-tailed Student's t-test and presented as mean ± SEM, n=4. *P <0.05, **P <0.01.

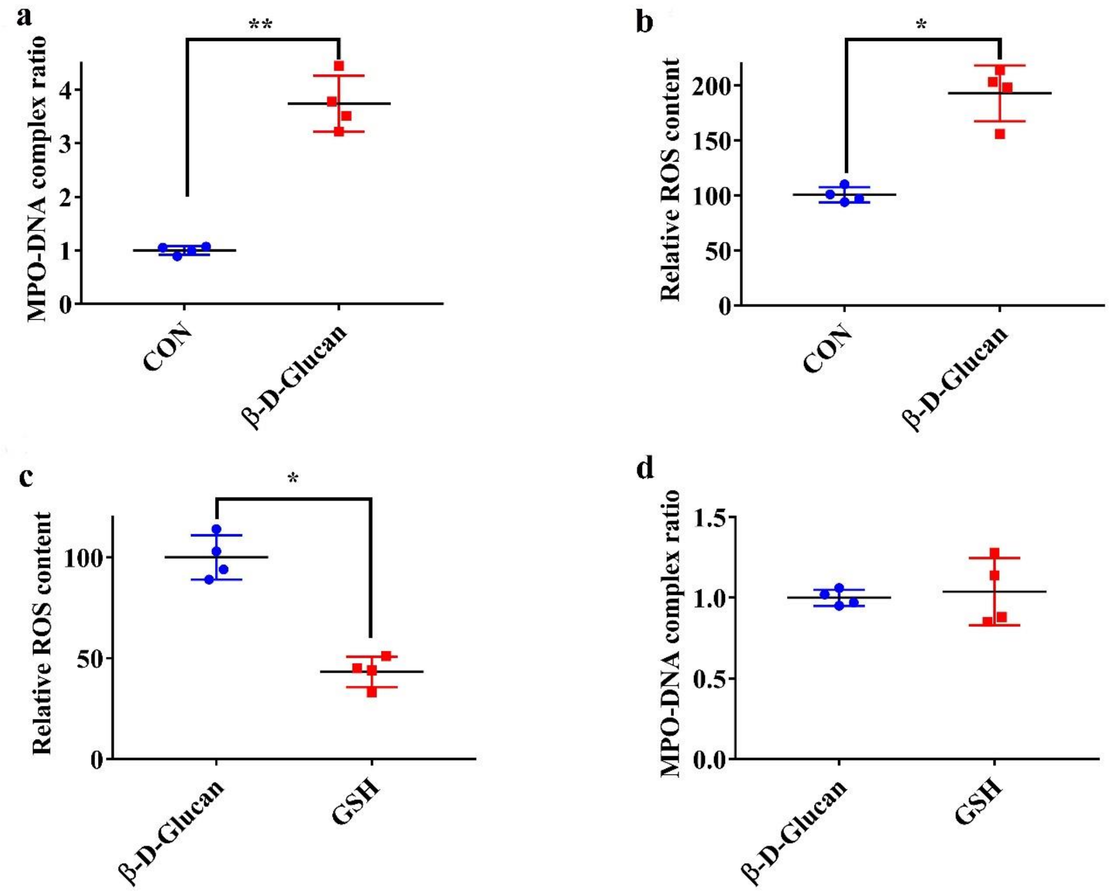

Supplemental Figure 7 Phosphocholine do not affect intestinal morphology, inflammation and tight junction function

(a) Intestinal morphology (Scale bar=200 μm); Relative gene expression of *TNF-α* (b) and *IL-1β* (c). Relative gene expression of *Tjp1* (d) and *Ocln* (e). Data were analyzed by two-tailed Student's t-test and presented as mean ± SEM, n=6. CONT, control mice; PC, mice were treated with 2.5 mol/L phosphocholine for 7 days.

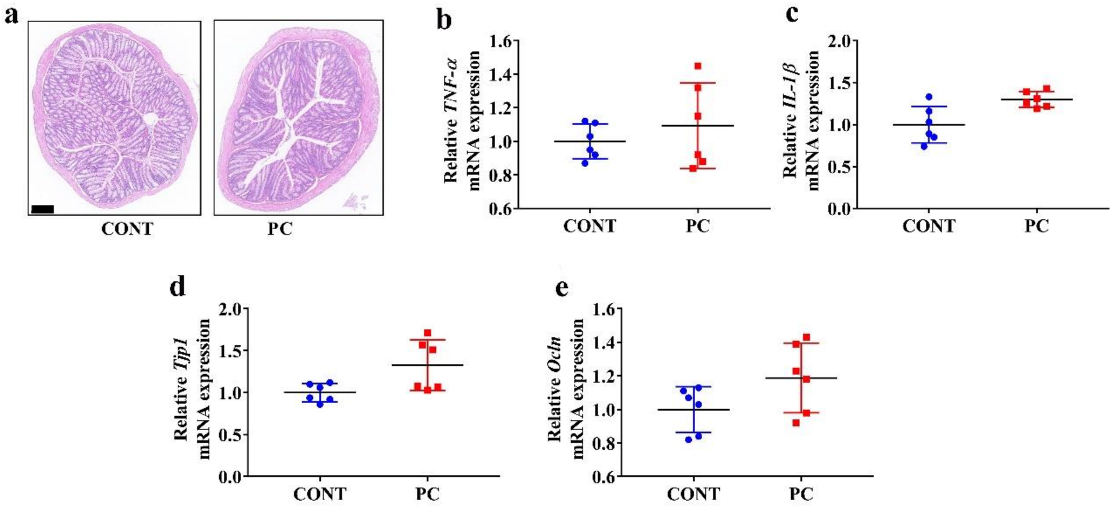

Supplemental Figure 8 Ca^{2+} level in the colon of mice treated with phosphocholine

Data were analyzed by two-tailed Student's t-test and presented as mean ± SEM, n=6.

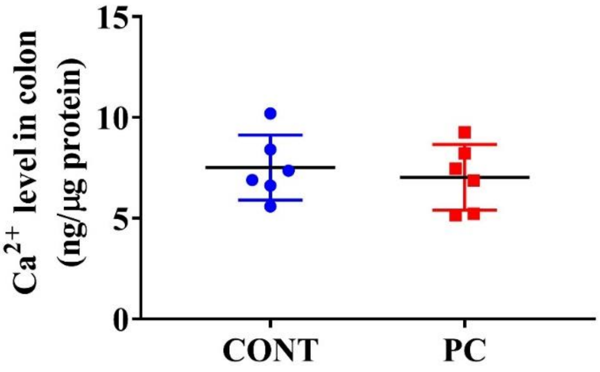

The full, uncropped western blots For Figure 6

GAPDH for Figure 6e and 6j

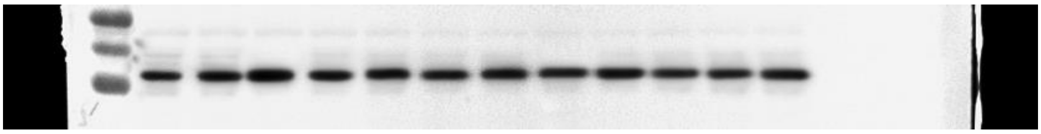

SLC9A3 for Figure 6e and 6j

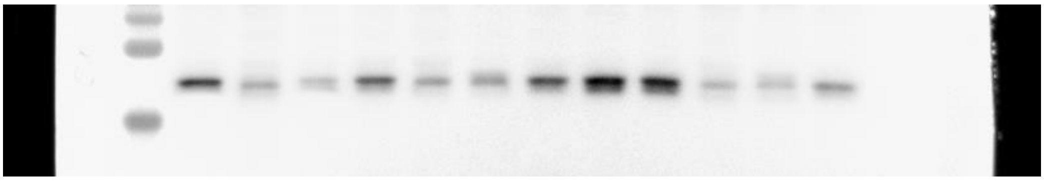

SLC5A1 for Figure 6e and 6j

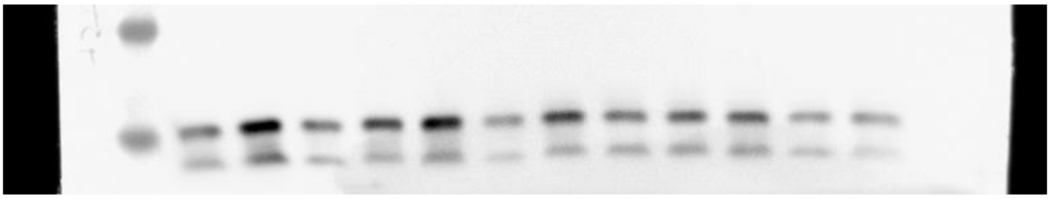

SLC26A3 for Figure 6e

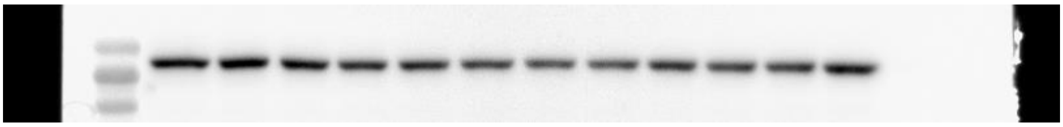

SLC26A3 for Figure 6j

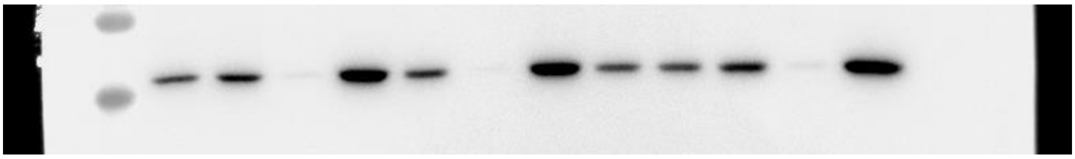

Supplement: Supplementary file 1 — SUPPLEMENTAL MATERIAL [file 41522_2023_433_MOESM1_ESM.pdf]
