# Supplementary material for: Colonic phosphocholine is correlated with Candida tropicalis and promotes diarrhea and pathogen clearance
Source: NPJ Biofilms Microbiomes. 2023 Sep 4;9:62. doi: 10.1038/s41522-023-00433-0 (PMC10477305; doi:10.1038/s41522-023-00433-0)
Supplement: Supplementary file 2 — Reporting Summary [file 41522_2023_433_MOESM2_ESM.pdf]

nature portfolio

Corresponding author(s): Xia Xiong, Jie Yin, and Yulong Yin

Last updated by author(s): Aug 1, 2023

Reporting Summary

Nature Portfolio wishes to improve the reproducibility of the work that we publish. This form provides structure for consistency and transparency in reporting. For further information on Nature Portfolio policies, see our Editorial Policies and the Editorial Policy Checklist.

Statistics

For all statistical analyses, confirm that the following items are present in the figure legend, table legend, main text, or Methods section.

n/a	Confirmed	
☐	☒	The exact sample size (*n*) for each experimental group/condition, given as a discrete number and unit of measurement
☐	☒	A statement on whether measurements were taken from distinct samples or whether the same sample was measured repeatedly
☐	☒	The statistical test(s) used AND whether they are one- or two-sided<br>*Only common tests should be described solely by name; describe more complex techniques in the Methods section.*
☒	☐	A description of all covariates tested
☐	☒	A description of any assumptions or corrections, such as tests of normality and adjustment for multiple comparisons
☐	☒	A full description of the statistical parameters including central tendency (e.g. means) or other basic estimates (e.g. regression coefficient) AND variation (e.g. standard deviation) or associated estimates of uncertainty (e.g. confidence intervals)
☒	☐	For null hypothesis testing, the test statistic (e.g. *F*, *t*, *r*) with confidence intervals, effect sizes, degrees of freedom and *P* value noted<br>*Give P values as exact values whenever suitable.*
☒	☐	For Bayesian analysis, information on the choice of priors and Markov chain Monte Carlo settings
☒	☐	For hierarchical and complex designs, identification of the appropriate level for tests and full reporting of outcomes
☒	☐	Estimates of effect sizes (e.g. Cohen's *d*, Pearson's *r*), indicating how they were calculated

Our web collection on statistics for biologists contains articles on many of the points above.

Software and code

Policy information about availability of computer code

Data collection	Roche LightCycler 480 II software used for collecting qPCR data.
Data analysis	Graphpad Prism 8.0 used for graphing data. SPSS Statistics software (version 18.0) used for data analysis.

For manuscripts utilizing custom algorithms or software that are central to the research but not yet described in published literature, software must be made available to editors and reviewers. We strongly encourage code deposition in a community repository (e.g. GitHub). See the Nature Portfolio guidelines for submitting code & software for further information.

Data

Policy information about availability of data

All manuscripts must include a data availability statement. This statement should provide the following information, where applicable:

- Accession codes, unique identifiers, or web links for publicly available datasets
- A description of any restrictions on data availability
- For clinical datasets or third party data, please ensure that the statement adheres to our policy

The ITS gene sequence data were deposited in the NCBI SRA database (https://www.ncbi.nlm.nih.gov/sra/) under accession number PRJNA891366. All data needed to evaluate the conclusions in the paper are presented in the paper and/or Supplementary Materials. Additional data related to this study may be requested from the corresponding author.

Research involving human participants, their data, or biological material

Policy information about studies with human participants or human data. See also policy information about sex, gender (identity/presentation), and sexual orientation and race, ethnicity and racism.

Reporting on sex and gender	not applied
Reporting on race, ethnicity, or other socially relevant groupings	not applied
Population characteristics	not applied
Recruitment	not applied
Ethics oversight	not applied

Note that full information on the approval of the study protocol must also be provided in the manuscript.

Field-specific reporting

Please select the one below that is the best fit for your research. If you are not sure, read the appropriate sections before making your selection.

☒ Life sciences ☐ Behavioural & social sciences ☐ Ecological, evolutionary & environmental sciences

For a reference copy of the document with all sections, see nature.com/documents/nr-reporting-summary-flat.pdf

Life sciences study design

All studies must disclose on these points even when the disclosure is negative.

Sample size	No statistical method was used to predetermine sample size. For animal studies, sample size was defined on the basis of our own past experience. Statistical significansce was obtained with these sample size. For animal studies, to minimize use of animals, only male mice were used for experiments in this study.
Data exclusions	No data were excluded in the current work when performing the final statistical analysis
Replication	All in vitro experiments were performed in triplicate unless specified in the figure legends. The detailed replication of each experiments has been provided in Figure Legend. All attempts at replication were successful.
Randomization	Animals were allocated randomly to each treatment group except that diarrheal piglets and healthy piglets were allocated according to diarrhea score. Different treatment groups were processed identically, and animals in different treatment groups were exposed to the same environment.
Blinding	The investigators were unaware of the experimental groups in all the quantifications.

Reporting for specific materials, systems and methods

We require information from authors about some types of materials, experimental systems and methods used in many studies. Here, indicate whether each material, system or method listed is relevant to your study. If you are not sure if a list item applies to your research, read the appropriate section before selecting a response.

Materials & experimental systems

n/a	Involved in the study	
☐	☒	Antibodies
☒	☐	Eukaryotic cell lines
☒	☐	Palaeontology and archaeology
☐	☒	Animals and other organisms
☒	☐	Clinical data
☒	☐	Dual use research of concern
☒	☐	Plants

Methods

n/a	Involved in the study	
☒	☐	ChIP-seq
☒	☐	Flow cytometry
☒	☐	MRI-based neuroimaging

Antibodies

Antibodies used	MPO (Abcam Cat#ab208670, RRID: AB_2864724), Histone H2A (Abcam Cat#ab177863, RRID: not found), Cit-H3 (Cell Signaling Technology Cat#97272, RRID, not found), Claudin-2 (Abcam, Cat#ab53032, RRID:AB_869174), Ki67 (Abcam Cat#ab15580, RRID:AB_443209), SLC9A3 (Bioss Cat#bs-8601R, RRID: AB_2928052), SLC5A1 (Bioss, Cat#bs-1128R, RRID: AB_10856441), SLC26A3 (Boster, Cat#A03335, RRID: not found), and GAPDH (Abcam Cat# ab8245, RRID: AB_2107448)
Validation	MPO, https://www.abcam.cn/products/primary-antibodies/myeloperoxidase-antibody-epr20257-ab208670.html<br>Histone H2A, https://www.abcam.cn/products/primary-antibodies/histone-h2ax-acetyl-k5--histone-h2a-acetyl-k5-antibody-epr17589-chip-grade-ab177863.html<br>Cit-H3, https://www.cellsignal.cn/products/primary-antibodies/citrullinated-histone-h3-arg17-e4o3f-rabbit-mab/97272?site-search-type=Products&N=4294956287&Ntt=97272&fromPage=plp&_requestid=1564602<br>Claudin-2, https://www.abcam.cn/products/primary-antibodies/claudin-2-antibody-ab53032.html<br>Ki67, https://www.abcam.cn/products/primary-antibodies/ki67-antibody-ab15580.html<br>SLC9A3, http://www.bioss.com.cn/prolook_03.asp?id=AF08169606015065&pro37=1<br>SLC5A1, http://www.bioss.com.cn/prolook_03.asp?id=AF08169606000761&pro37=1<br>SLC26A3, https://www.boster.com.cn/home/product/anti-dra-slc26a3-antibody_a03335.html<br>GAPDH, https://www.abcam.cn/products/primary-antibodies/gapdh-antibody-6c5-loading-control-ab8245.html

Animals and other research organisms

Policy information about studies involving animals; ARRIVE guidelines recommended for reporting animal research, and Sex and Gender in Research

Laboratory animals	Dectin-1 knockout mice with a C57BL/6 background and wild-type littermates aged 8-10 weeks were used; Duroc ×Landrace × Yorkshire piglets weaned at the age of 21 days were used.
Wild animals	not applied
Reporting on sex	Male and female piglets were used and we found that sex did affect the difference between diarrheal and healthy control piglets. Thus, further study with piglets and mice used male animals only.
Field-collected samples	not applied
Ethics oversight	All mice were housed under specific pathogen-free conditions) and piglets were cared for at the Institute of Subtropical Agriculture, Chinese Academy of Sciences. Animal procedures were approved by the Protocol Management and Review Committee of the Institute of Subtropical Agriculture, Chinese Academy of Sciences (ISA2021091).

Note that full information on the approval of the study protocol must also be provided in the manuscript.
